# Trust Information-Based Privacy Architecture for Ubiquitous Health

**DOI:** 10.2196/mhealth.2731

**Published:** 2013-10-08

**Authors:** Pekka Sakari Ruotsalainen, Bernd Blobel, Antto Seppälä, Pirkko Nykänen

**Affiliations:** ^1^School of Information SciencesCenter for Information and SystemsUniversity of TampereTampereFinland; ^2^eHealth Competence CenterUniversity Hospital RegensburgUniversity of RegensburgRegensburgGermany

**Keywords:** ubiquitous health, privacy, computational trust, policy, context-awareness

## Abstract

**Background:**

Ubiquitous health is defined as a dynamic network of interconnected systems that offers health services independent of time and location to a data subject (DS). The network takes place in open and unsecure information space. It is created and managed by the DS who sets rules that regulate the way personal health information is collected and used. Compared to health care, it is impossible in ubiquitous health to assume the existence of a priori trust between the DS and service providers and to produce privacy using static security services. In ubiquitous health features, business goals and regulations systems followed often remain unknown. Furthermore, health care-specific regulations do not rule the ways health data is processed and shared. To be successful, ubiquitous health requires novel privacy architecture.

**Objective:**

The goal of this study was to develop a privacy management architecture that helps the DS to create and dynamically manage the network and to maintain information privacy. The architecture should enable the DS to dynamically define service and system-specific rules that regulate the way subject data is processed. The architecture should provide to the DS reliable trust information about systems and assist in the formulation of privacy policies. Furthermore, the architecture should give feedback upon how systems follow the policies of DS and offer protection against privacy and trust threats existing in ubiquitous environments.

**Methods:**

A sequential method that combines methodologies used in system theory, systems engineering, requirement analysis, and system design was used in the study. In the first phase, principles, trust and privacy models, and viewpoints were selected. Thereafter, functional requirements and services were developed on the basis of a careful analysis of existing research published in journals and conference proceedings. Based on principles, models, and requirements, architectural components and their interconnections were developed using system analysis.

**Results:**

The architecture mimics the way humans use trust information in decision making, and enables the DS to design system-specific privacy policies using computational trust information that is based on systems’ measured features. The trust attributes that were developed describe the level systems for support awareness and transparency, and how they follow general and domain-specific regulations and laws. The monitoring component of the architecture offers dynamic feedback concerning how the system enforces the polices of DS.

**Conclusions:**

The privacy management architecture developed in this study enables the DS to dynamically manage information privacy in ubiquitous health and to define individual policies for all systems considering their trust value and corresponding attributes. The DS can also set policies for secondary use and reuse of health information. The architecture offers protection against privacy threats existing in ubiquitous environments. Although the architecture is targeted to ubiquitous health, it can easily be modified to other ubiquitous applications.

## Introduction

### Overview

Both ubiquitous health and pervasive health are terms that describe a new business model (these terms have been used in many papers synonymously). Similarly to health care, its goal is to make health services available to everyone, but many of its features separate it from health care [1]. According to Ruotsalainen et al, ubiquitous health is a metasystem that is a dynamic network of interconnected systems offering health services to a data subject (DS) in an unsecure information space [1]. Contrary to health care where the services are defined by health professionals, in ubiquitous health, the DS creates the network, selects the systems, and sets rules (policies) that regulate how and by whom the DS’ health information is used and shared. In ubiquitous health, the existence of predefined trust between the DS and systems cannot be assumed, and systems’ features, their business goals, and regulation systems followed are often unknown. Furthermore, health care-specific regulations do not rule the ways health data is processed and shared [1]. It is evident that ubiquitous health features generate privacy and trustworthiness challenges that should be solved to make it successful.

Privacy is a complex, personal, and situation-depending concept that can be interpreted in various ways [2]. Westin defined privacy as “the claim of an individual to determine what information about himself or herself should be known to others and what uses will be made of it by others” [3]. Privacy is also a human right that is protected by international directives and constitutions. Privacy protection approaches aim at hiding user’s identity and/or some part of the personal identifiable information (PII), whereas privacy management offers transparency to the DS concerning the collection and processing of PII.

Trust can be understood as the subjectively perceived probability by a DS that a system will perform an action before the DS can monitor it [4]. It indicates uncertainty about the features of communication partners [5,6]. Trust is also context-dependent and the ways it is formulated vary, for example, it can be based on the recommendation received from others, it can be reputation-based, or it may be a subjective degree of belief of others [7,8].

Privacy and trust are interrelated concepts, that is, “data disclosure means loss of privacy, but an increased level of trustworthiness reduces the need for privacy” [1]. The DS interest is to get maximum benefit from services and at the same time to minimize the loss of privacy.

In health care, internationally accepted principles, good practice rules, and domain-specific legislation define patient’s rights and service providers’ responsibilities. Health care-specific legislation also states how patient’s privacy must be protected [1]. Researchers have started to develop such kind of principles for ubiquitous health. Ruotsalainen et al have developed the THEWS (Trusted eHealth and eWelfare Space) principles for trustworthy ubiquitous health. The THEWS principles state that the DS possesses the right [1]: to verify the trustworthiness any system that collects or processes his or her personal health information (PHI). Principles state that DS should also have the right for controlling the processing of PHI, both inside the systems and between them. DS should define personal privacy policies, which regulate how his or her health data is collected, processed, disclosed, shared, stored, or destroyed. The principles also require the DS to be aware of all events, situations, and contexts where his or her health data is collected, processed, stored, and disclosed.

Furthermore, systems and stakeholders have the responsibility to publish information needed for trust verification and support openness and transparency of data processing.

Ubiquitous health features and its ubiquitous environment suggest that trustworthiness and privacy are real concerns [9,10]. In ubiquitous health, it is difficult to understand the processing of data inside the systems [11], as systems do not always perform in accordance with their policies, and the privacy preferences of DS might conflict with the business objectives of the system [12]. As a result, the DS cannot assume that the existing legal framework guarantees the processing of PHI lawfully and according to the rules proposed by him or her [13,14]. In addition, DS also cannot assume that systems have implemented security rules and functional privacy requirements derived from laws and standards [1,15]. A big challenge in ubiquitous health is that different stakeholders (eg, systems, customers, third parties, and regulators) can have their own privacy policies.

Here we hypothesize that in order to be successful, ubiquitous health requires trustworthiness and privacy management made by the DS. Without these two features, DS will not dare to use its services. Furthermore, the architecture supporting ubiquitous health should fulfill the THEWS principles presented above. As traditional security and trust mechanisms used in today’s health care information systems may not provide adequate security and privacy in ubiquitous health [1,2,16], a novel architecture is required.

### Prior Work

The development of ubiquitous systems and the growing use of ubiquitous computing have raised the following question: What kind of trust and privacy models, services, and architectures offers acceptable level of privacy and trustworthiness?

### Trust Models

Trust models such as belief, organizational trust, dispositional trust, recommended trust, and direct trust have been proposed for pervasive systems [8,17,18]. Dispositional trust describes the general trusting attitude of the trustor [17]. Direct trust is derived from the outcomes of interactions with peers [19]. In recommended trust, an agent makes a recommendation based on the beliefs that other entity is trustworthy at certain degree. Organizational or institution-based trust is based on the perceived properties of, or the reliance placed on, a system or institution [7]. Reputation is a recommended rating based on the opinions of others [8]. All of them are situational, that is, the amount of trust that a DS experiences depends dynamically on situation and service-specific trust features [20,21].

A trust is typically based on the trustor’s characteristics such as ability, integrity, and benevolence and should not be a blind guess [5]. It is expressed either by value, rating, or ranking or as probability or belief [22]. Trust attributes such as integrity, motivation, competence, and predictability are proposed to measure the confidence level [23]. Attributes proposed by Hussin include trustee’s identifier, certificate, ability, predictability, trustee’s privacy policy, legal requirements, and system’s properties such as transparency, authenticity, confidentiality, and nonrepudiation [24]. Researchers have developed mathematical methods such as Bayesian probability, Beta probability, maximum likelihood, game theory, weighted arithmetic means, and average of weighted recommendations to measure the degree of belief or recommended trust [25-27]. Trust degree can also be measured from interaction frequencies between trustor and trustee [28], or from context-dependent direct and indirect recommendations collected from selected users [19].

In contrast to belief and recommended trust, computational trust built on abstractions of human concept of trust has been proposed by researchers [25,29]. Within ubiquitous computing, computational trust means automation of decisions in the presence of unknown, uncontrollable, and possibly harmful agents [29]. Computational trust value has been calculated using trustor’s experience, recommendations, interactions, knowledge, measurements, distance, and density of events [13,25,28,30,31]. Service level agreements, contractual agreements, reputation based on the brand’s name, trust manifesto, trust negotiation, exchanging and evaluating credentials, and recommendations made by a trust authority (TA) are also widely used in commercial eServices [32,33].

The aforementioned trust models have noticeable weaknesses in ubiquitous environment. Recommendations are unreliable because they are based on unsecure opinions. It is difficult to force everyone to accept certificates or common TA, and many virtual organizations do not have connection to it. A common ontology that is required for successful negotiation and calculation of trust attributes seldom exists. Trust manifesto assumes that the DS blindly trusts that service providers will deliver their promises. Furthermore, the reliability of reputations is difficult to measure, and credentials are difficult to evaluate [25].

### Privacy Models and Formula

Many privacy models developed by researchers are useful in ubiquitous environment. Lederer et al proposed a model of situational faces [34]. The model proposed by Hong et al uses control and feedback [10]. The model suggested by Friedwald et al included actors, environment, activity, information flow, control level, and enabling technology [35]. Adams and Sasse look at privacy as preferences and constraints, and use a computer-understandable language for expressing them [36]. Jiang and Landay used an information space model [37], and Kapadia et al applied virtual walls for privacy management [38]. Diaz et al proposed entropy as measure of privacy level [39].

Privacy management model proposed by Lederer et al combined Adams’s perceptual model and Lessing’s societal privacy models [40,41]. In the model by Lederer et al, a preferred privacy level depends on legislation, market features, norms, technology used, nature of personal information disclosed, contextual features, information sensitivity, characteristics of information user, and expected cost-benefit ratio. A limitation of this model is that its variables are qualitative and abstract.

### Trust and Privacy Technologies and Solutions

Numerous trust and privacy technologies have been proposed for ubiquitous systems. In Gray’s solution, the trust is based on the belief of a person that systems have implemented proper de-identification structures and safeguards. It also includes a compliance checker and a trust value calculator [42]. PoliCyMaker, KeyNote, Simple Public Key Infrastructure, and Pretty Good Privacy solutions use credentials [43]. The Trust-X approach by Bertino et al uses digital credentials, which are iteratively disclosed and verified [32]. Becerra et al proposed intelligent agents to evaluate which other agents can be trusted [23]. According to the Skopik’s approach, rule-based trust interpretation takes into account the subjective nature of trust [44]. Joshi et al noted that it is possible to make security and privacy decisions based on trust attributes [45].

Computational trust is either based on direct measurements, observed (monitored) features, or past experiences [46]. In ubiquitous environment, successful monitoring requires common ontology and measurable indicators [22]. The trust manager architecture proposed by Salah et al collects trust aspects for calculator that computes a trust score. The architecture also includes recommendation manager, monitor services, context provider, log service, and policy manager [47]. In the EnCoRe architecture, the TA keeps track of promises, manages decryption keys, discloses them, and verifies systems properties [48]. Thereby, the customer should trust on the system’s released willingness to fulfill the personal policies of DS.

Privacy is often protected by using privacy enhancement solutions such as data filtering and minimization, anonymization, and adding noise to disclosed information (eg, data hashing, cloaking, blurring, and identity hiding) [41,49]. In metadata approaches, privacy policies can be injected to application, tagged to the metadata, or added to the database or an active agent [50]. Berghe and Schunter’s “privacy injector” adds privacy rules to existing applications [11]. The EnCoRe architecture uses the sticky policy paradigm where the DS can stick machine-readable rules to the data before it is disclosed [48]. Metadata can include embedded (active) code that enables self-destruction (apoptosis) in the case the environment is not trusted [51]. Apoptosis can also be context- or situation-aware (ie, programmed death) [52]. As per Pallapa et al, active privacy metadata dynamically controls the transparency of data in a context [53].

Other solutions also exist for privacy protection. Kapadia et al created a virtual personal space (a room) to control information flow through its “walls” [38]. In the PICOS platform from Kahl et al, a privacy advisor helps the DS to create own policies [54]. In the United States, a flexible approach that uses privacy and security labels is under development. In this standardized solution, PHI is segmented and security and privacy labels are bound to those segments [55].

In pervasive systems, privacy requirements are typically expressed as policies that are context-dependent. Policies define what is permitted or prohibited, and which are permitted actions [45]. From the DS viewpoint, policy can be understood as a statement (rules) about how a certain system should behave [56]. Policies are typically published in the form of credentials or metadata, and rules are expressed using policy language [33]. The successful use of policies requires policy matching, mismatch notification, policy lifecycle management, risk analysis, regulatory compliance checking, and possibility to model privacy regulations [48,57]. It is also necessary that the DS can enforce personal polices [58]. Policies should also be checked for ontological compatibility [59].

The increasing use of the Internet, peer-to-peer systems, multi-agent systems, and social networks has been main drivers for discussed privacy and trust models and solutions. Unfortunately, most of them are focused on one feature (eg, encryption or context). Ubiquitous health requires much wider approach. Like Bryce et al, we also state that pervasive systems require an architecture that combines dynamic privacy policies, a priori trust validation, privacy management, and a posteriori measurement (ie, feedback) what systems are doing [2]. Regulatory compliance is also needed.

In this paper, we propose a novel privacy management architecture for ubiquitous health. As ubiquitous health is a new concept without widely accepted principles and privacy and trust models, it is necessary to select on which principles and models the architecture is based. THEWS principles, as previously presented, have been selected by the authors on the basis of the architecture, that is, the architecture should be compliant with them. The solution should take into account features of ubiquitous health and enable the DS to dynamically manage the privacy by defining system-specific privacy policies. The architecture should mimic the way humans use trust information in creation of personal policies. The architecture should also offer protection against many known privacy threats existing in ubiquitous environment.

## Methods

**Figure 1:**
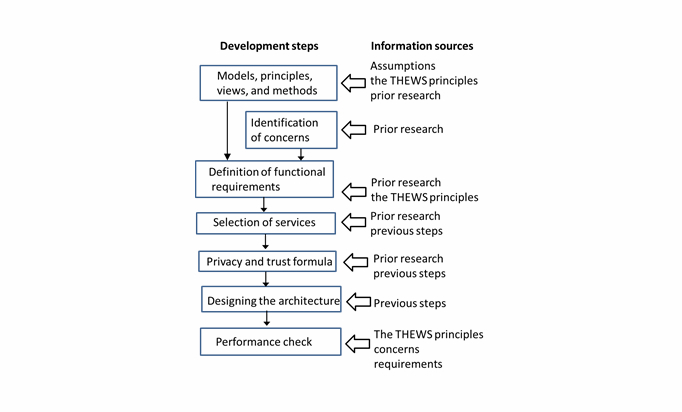
Method for the development of the THEWS architecture.

From system theory and systems engineering perspectives, ubiquitous health is a metasystem that is characterized by its structure, its function/behavior, and how its interrelated components are composed in an ordered way. Instead of creating artificial scenarios or making quantitative privacy risk/threat analysis, a more system-oriented sequential method that combines methodologies used in systems engineering, requirement analysis, and system design is used (Figure 1).

The method used in this study includes the following steps: definition of basic requirements; selection of values, privacy and trust models, and views; identification of concerns; definition of functional requirements; selection of services; developing privacy and trust formula; and designing the architecture. Finally, it is checked how the architecture meets purposes and requirements for which it has been intended.

On the background of processing of health information stay ethical values and codes, principles, and common rules. Selection of these features has also strong impact on the architecture and its services. For some environments (eg, health care), widely accepted codes and rules already exist; however, this is not the case in ubiquitous health. Therefore, the first step is to select privacy and trust models and approaches that are in line with principles and without noticeable weaknesses. This is achieved by carefully analyzing existing research published in journals, conference proceedings, and standards documents. Similarly, identification of concerns and definition of functional requirements are also done. Finally, the architecture combines selected services in such a way that principles and requirements are fulfilled.

In this paper, privacy and trust needs are examined from the DS’s viewpoint. Other views are not discussed. To reduce the complexity, only components that are relevant for the privacy management needs of the DS are included in the architecture.

## Results

### General Overview

Ruotsalainen et al have noted that privacy rules in ubiquitous health are based on trust [1]. Therefore, privacy and trust models selected should take into consideration features of ubiquitous health, trust and privacy aspects of systems offering health services, regulatory requirements, and the DS’s privacy needs. The asymmetric relationship between systems providing health services and the DS should also be considered (ie, the DS seldom has the power to force a system to put personal rules into effect). Furthermore, in practice, the DS has no tools to make personal observations of systems’ internal security and privacy features and policies [51,60].

### Principles, Models, and Views

In spite that privacy is widely accepted as human right (value), different privacy models do exist in real life. Regulatory and self-regulatory models are widely used [15]. Privacy can also be considered as personal property [20]. Regulatory model is insufficient in ubiquitous environments [13], and self-regulation made by business community gives systems as stronger partner much freedom to set rules [15]. Because in ubiquitous health the DS has the right to set personal rules to regulate and control his or her health information, self-regulation model that uses privacy as the DS’s personal property has been selected for the architecture.

Suitability of widely used privacy protection and management approaches in the context of ubiquitous health is shown in Table 1. Based on Table 1 and the fact that pervasive systems require dynamic and context-aware privacy management [46], the foremost privacy approach for ubiquitous health is privacy management that uses context- and content-aware policies and supports transparency and regulatory compliance.

Trustworthy ubiquitous health requires that used trust model enables the DS to work out the level of trustworthiness of systems. Characteristics and weaknesses of widely used trust models in regard to features of ubiquitous health are shown in Table 2. As a result, trust in ubiquitous health cannot be based on the belief or reputation, and the DS usually does not have a right to verify recommended trust. Credentials typically assume that Hobson choice and privacy labels have inappropriate granularity. Although some researchers assume that the protection power of laws is sufficient and certification offers acceptable level of trust [12], the regulations and certificates are found to be insufficient in ubiquitous health.

Computational trust that is based on systems’ measurable or observed properties can offer reasonable information to the DS in designing personal privacy policies [25]. The limitation that the information content of a single trust value is too low for policy formulation [61] can be overcome by using additional system-specific attributes. Therefore, computational organizational trust with attributes is selected as the trust model for ubiquitous health.

From the DS viewpoint, the architecture should mimic humans’ ways to design policies, support more rational choices than intuition, and give feedback to the DS. Louviere’s stated customer choice method fulfills these requirements by including awareness, learning, evaluation and comparison, preference formulation, and choice and post-choice [62]; hence, it is selected for the method that the DS uses in the formulation of privacy policies.

**Table 1:** Suitability of common privacy protection and management approaches for ubiquitous health.

Approach	Suitability
Privacy protection using security services (eg, authentication, authorization, and access control)	Security cannot offer reasonable level of privacy in ubiquitous health. Access control alone is insufficient. The DS is not familiar and cannot control authorization rules used inside a system
Privacy control by hiding the DS’s identity	Health care and health services require the knowledge of the DS’s identity
Delegation approach	Delegation requires knowledge to whom the DS delegates access rights. Systems specifically do not publish this kind of information to the DS
Privacy labels	Rules deployed in a label might be inadequate and in conflict with the DS policy that may or could not be specified in labels
Privacy management using context- and content-aware policies	Supports dynamic policies, but requires computer-understandable policy language. Common ontology, ontology harmonization (matching, mapping, etc.), or reasoning is needed
Metadata approach	All systems do not accept injected or active code
Data filtering and adding noise to data	Health services require large amount of PHI for correct and effective services, as incomplete PHI can lead to wrong decisions or prevent the use of services

**Table 2:** Characteristics and weaknesses of common trust models.

Model	Characteristics and weaknesses in ubiquitous health
Dispositional trust and recommended trust	Characteristics: Based on belief, attitude, or others’ opinions (recommendations)
	Weakness: Recommendations are unreliable and based on unsecure opinions. It is difficult or even impossible to check the reliability of others’ recommendations
Blind trust	Characteristics: Based on belief or attitude that organization has implemented sufficient safeguards
	Weakness: Does not guarantee trustworthiness
Predefined trust	Characteristics: Based on assumption that an organization has implemented required regulatory services
	Weakness: Static model. Unsuitable for dynamic environments.
Trust label	Characteristics: Based on organizational or personal labels
	Weakness: Inappropriate granularity and insufficient consideration of dynamic contextual conditions
Trust manifesto	Characteristics: Based on assurance of service provider
	Weakness: Based on belief or attitude. The DS should blindly trust
Reputation	Characteristics: Based on subjective opinions of others
	Weakness: The reliability of reputations is difficult to measure
Computational trust	Characteristics: Based on system’s measured or observed features
	Weakness: A simple trust value or rank might offer insufficient information for the DS in designing personal policies
Risk- and threat-based models	Characteristics: Based on risk or threat assessment
	Weakness: Difficult or even impossible to measure personal privacy risks
Trust management using credentials	Characteristics: Based on credentials issued by authorities. It is targeted to create trust between organizations
	Weakness: Credentials are static. Difficult to evaluate and require a network of trusted authorities. It is difficult to force everyone and virtual systems to accept credentials or a TA

### Identification of Concerns

Typical stakeholders in ubiquitous health are the DS, health service providers, other organizations, and secondary users. Different stakeholders have different concerns [1]. This paper is focused to the DS concerns. The main concerns of the DS are as follows: (1) how trustworthy the system is, (2) why is lack of awareness and transparency in data collection and processing, (3) who is using the data inside a system, (4) how to guarantee that data is processed lawfully, and (5) according to the DS’s policies, how to prevent post-release of data and control unnecessary secondary use.

### Functional Requirements

Derived from previously mentioned assumptions and selections and the proposals made by other researchers, the architecture should identify the following functional requirements. The architecture should offer tools for the DS to define purposes of data collection, express computer-understandable rules regarding the sensitivity of data elements, design protection needed, rule how long data is stored, and which data is disclosed and for what purposes [14,48].

The architecture should support dynamic content-, context-, and purpose-aware privacy management. It should also offer to the DS system-specific computational trust information with attributes that describe systems’ features, infrastructures, policies, and relations in advance. Humans’ way to design policies, to support more rational choices than intuition, and to give feedback should need to be mimicked. The architecture must be compliance with Louviere’s stated customer choice method. It should support situations where the DS discloses PHI and where data collection or disclosure is made autonomously by a system. The architecture also enables the DS to be aware of data-processing events, and to set policies regulate the secondary use and reuse of PHI.

### Trust and Privacy Services

Services of the architecture should fulfill above-mentioned requirements, and take into account expected concerns. Trust and privacy services selected for the THEWS architecture are shown in Table 3.

**Table 3:** Trusts and privacy services for the THEWS architecture.

Concern/Function	Service
System’s trustworthiness	Trust calculation service
	Context service
	Identification service
	Trust interpreter service
The DS’s information autonomy	Decision support service
	Policy-binding service
Awareness and transparency	Monitoring, trust calculation, and notification services
The use of PHI inside the system	Monitoring and notification services
Does the system use PHI according to the DS’s policies	Monitoring and notification services
Choice and secondary use and post-release of PHI	Policy-binding service
	Metadata (eg, sticky policy or active code for apoptosis)
Designing privacy policies and comparison and preference formulation	Decision support service
Policy formulation and post-choice and new policy creation	Policy management service
	Policy assistant service
	Ontology service
System’s features and relations	Trust calculation service
Feedback and alarm or conflict notice	Monitoring service
Learning	Trust interpreter and policy assistance services

### Privacy and Trust Formula

The THEWS principles and functional requirements determine that the DS can use trust information in the formulation of privacy policies [1]. The following formula has been developed to illustrate how trust information, privacy variables, and privacy policy are related:

*Privacy_policy=f(TI, IS, SE, PU)*

In this formula, TI refers to *trust_information* offered by the architecture to the DS. IS, SE, and PU are privacy variables proposed by Lederer [40]. IS refers to the sensitivity of the data, SE describes the situation where information is used, and PU defines the purpose of data collection or use.

To avoid the drawback of a single calculated trust value and to enable attribute-based creation of personal policies [61], the following trust information formula was developed:

*Trust_information=Trust_value+Trust_feature_vector*

*Trust_feature_vector* gives the system- and environment-specific information to the DS about systems’ regulatory compliance and their willingness to follow the DS’s policies and support openness. Slightly modified trust attributes originally proposed by Hussin et al have been selected for trust value calculation [24]:

*Trust_value=(E, T, P, PO, Pre, Tran, Ab)*

where E represents domain specific environmental factors such as legal requirements and system’s contextual features. T represents the type of service provider’s organization (eg, public health care provider, private health service provider, Internet service provider). P (properties) consists of systems architectural and technological aspects and PO is system’s privacy policy. Predictability (Pre), transparency (Tran), and ability (Ab) are different parameters that can be calculated from the system’s past history or by direct measurements. For *Trust_feature_vector*, the following formula was developed:

*Trust_feature_vector=(DGD, DRB, SPO, DSP, ASP, CD, ATV, AUT, RP, PBL, DSA)*

where DGD and DRB describe the level of system’s regulatory compliance. The DGD is the degree of data processing made by the system in compliance with international privacy protection directives. The DRB is the degree of data processing performed by the system compliant with health care-specific laws and rules. SPO and RP are parameters that are related to openness. SPO informs if the system has made its privacy policies openly available, and RP tells the status if the system has published its relationships. DSP, ASP, ATV, and AUT are willingness parameters. DSP describes the degree by which the system follows its own privacy policies. ASP informs that the system either enables or rejects the DS to inject personal policies to PHI collected or processed by the system. The ATV expresses whether the system accepts external monitoring of events related to the processing of PHI, and AUT tells whether the system enables external access to its audit trails. The PBL and CD are trustworthiness parameters. CD informs whether the system has been certified, and PBL informs about the position of the system on the blacklist. The DSA is an optional attribute that can be defined by the DS. For DGD and DRB, a linear scale (0...1) is used, whereas all others attributes have only binary values. In case of no or insufficient data, the attribute value is zero.

Using proposed *Trust_information*, the DS can predict system’s willingness or ability to process PHI legally and follow rules set by the DS. The *Trust_information* informs the DS about how much it can trust on a system, how system’s policy and technical architecture look like, and to what extent system’s policy is compliant with domain-specific regulations and laws. If needed, the DS can use attributes to mark a system untrusted (eg, in the case it will not publish its policies nor would accept monitoring). Most attributes can be calculated from information the system has, or should have, published; however, some attributes might require direct observations. Attributes such as DSP can be calculated from the system’s past history.

### The THEWS Architecture

**Figure 2:**
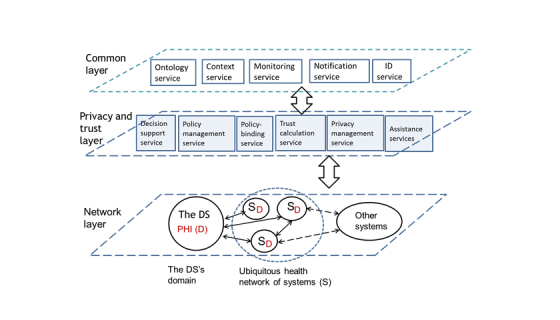
The framework model for the THEWS architecture.

A layered framework model that describes trust and privacy services of the THEWS architecture is shown in Figure 2. The top layer of the model consists of common services that are offered to all stakeholders. The middle layer includes privacy and trust services needed. Ubiquitous health, stakeholders, other users, and PHI are located in the lowest layer (ie, network layer).

As it is difficult or even impossible for the DS to evaluate the trustworthiness of systems, an independent agent, the trust calculator (TC), is used for this task. The role of TC is not to make trust decisions. Similar to HL7 Privacy, Access and Security Services architecture, the TC should be understood as an information point that sends trust information to the DS [55].

The TC calculates *Trust_information* (ie, *Trust_value* and related *Trust_feature_vector*) by using the information that system has published, and available contextual data, system’s measured or monitored features, and system’s past history. It also detects malicious or fake systems by using information obtained from context and monitoring services. Two assistance services are offered to the DS: (1) trust interpreter and (2) policy assistance service. The DS can use the trust interpretation to understand the meaning of received *Trust_information*.

The context service collects systems’ contextual data, interprets it, and makes it available to TC and DS, using ontologies. The DS deploys policy management, policy-binding, policy assistance, and decision support services in policy formulation.

The monitoring service offers feedback, reduces risk, and recognizes policy conflicts. It records and assesses how a system in real life processes PHI. It recognizes policy conflicts and alarms the TC and the DS of possible malicious or illegal use of PHI. The notification service works as communication and transparency tool between the DS, systems and services. Using this service, the DS expresses personal policies to systems that in turn publish their policies and relations.

**Figure 3:**
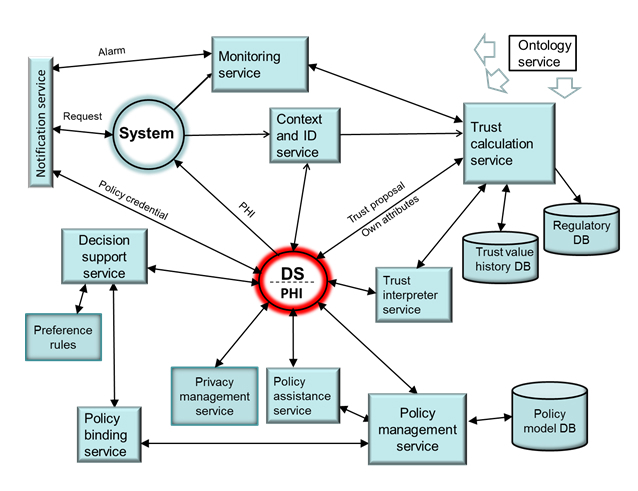
The interconnection of privacy and trust services in the THEWS architecture.

An architectural model describing the interconnection of the THEWS services is shown in Figure 3. In the architecture, the policy formulation is a decision-making process, where the DS chooses privacy rules, privacy management services, and the amount of PHI he or she wants to trade in according to expected service benefits. The selected rules and services depend on privacy needs, *Trust_information*, and the purpose of data request. Typical privacy management services that can be activated before data disclosure are encryption, anonymization, and data filtering. The DS may also inject policies and/or active code to the metadata.

The THEWS architecture not only fulfils the THEWS requirements but also offers protection against many of the known privacy threats existing in pervasive systems as shown in Table 4.

**Table 4:** The THEWS architecture approach for the challenges existing in pervasive systems.

Challenges and threats	THEWS approach
Pervasive systems are dynamic in nature (eg, ad hoc networks) where static rules and privacy services will not work	Dynamic rules and services are used
	Dynamic creation and management of the DS’s privacy service portfolio
No predefined trust	Dynamic trust calculation based on systems’ measured properties
The need of PII is dynamic and purposes are unpredictable	Dynamic context-aware polices support ad hoc purposes
Organizations do not always follow their own policies, and laws will be ineffective without sufficient control and penalties	The way systems process PHI is dynamically monitored, and the regulatory compliance is checked
Users want to control how systems use PII	The DS define system-specific policies that rule the use, storing, and sharing of PHI
It is difficult to know what is the actual privacy status of an enterprise (ie, what data and under what policy)	Status and policies are inspected and informed dynamically to the DS
It is difficult to know how data has been used inside the enterprise	The monitoring service can check internal use
Relationships between systems can be unknown	Systems must publish their relations
All service providers do not use certificates	Trustworthiness is not based on certificates
Selection of service provider needs trust and/or reputation	The TC offers calculated trust value and trust attributes to the DS
	Reputation is not used
Determining of systems’ trustworthiness is challenging	The TC calculates trust using direct measurements
	The monitoring service gives feedback to the TC
Which action the DS must take in the case of privacy breach?	The TC and/or monitoring service inform the DS of privacy breaches
	The DS can change policy dynamically
How to guarantee that data is processed lawfully and according to the DS’s policies	Trust attributes offer required information
	The monitoring service notifies misuse
Lack of awareness	Systems must publish their rules and relationships
	Awareness by monitoring service
How to know what actions are permitted or forbidden in a context and what actions must be performed?	The DS defines personal context-aware rules
How we can trust on systems privacy notices (or privacy manifesto)?	Privacy notice/manifesto is not used
Threats caused by surveillance, identity theft, or malicious attacks	Communication platform and systems must implement reasonable safeguards
Code of conduct, legal framework, and accreditation of centers will not guarantee trustworthiness	Those models are not used
Consent does not guarantee adequate protection	Consent is only one possible item in the policy
Anonymization such as “we know” will not guarantee adequate protection	Anonymization is only a value-added service
Secondary use of PII must be monitored	Monitoring service
Citizens need audit information	The monitoring service assesses the audit log and informs findings to the DS
	The TC can maintain a list of untrusted or malicious systems
Data requestors can have subjective views of trust	The TC defines the used trust ontology
How can we manage trust for systems with incomplete credentials?	Credentials are not used

## Discussion

In this study, novel privacy architecture is developed for ubiquitous health. It enables the DS to ensure and manage information privacy by choosing personal context-aware privacy policies for each system with the help of computational trust information that includes a trust value and system-specific trust attributes. The architecture combines many trust and privacy services proposed by researchers for pervasive systems such as trust calculation and interpretation, policy management, policy assistance, policy binding and design, and context services and monitoring. The architecture goes far beyond the security services with traditional access control used in health care, and it also illustrates how the THEWS principles can be realized. Furthermore, the architecture offers protections against many privacy threats caused by ubiquitous computing and unsecure environment. Instead of continuous validation of systems’ trustworthiness, the architecture monitors functioning of the systems, detects and informs the DS of policy conflicts and data misuse, and thereby enables the DS to dynamically change policies.

Contrary to a widely used trust manifesto that is based on incomplete, insufficient, or inconclusive information [33] or a single trust value that offers only Hobson’s choice to the DS, the architecture gives information to the DS that indicates the level of transparency and openness of a system, how system follows health-specific privacy rules and regulations, and how mature the system is to accept the DS’s policies. Using this information and policy assistance, decision support, and policy-binding services of the architecture, the DS can construct context- and content-dependent policy profiles and assign them to systems. The architecture is user-friendly, and there is no need to interactively calculate the trust value against the DS’s dynamic privacy needs.

For all pervasive systems, some of the unsolved privacy challenges are as follows: (1) How to prevent data from being collected and used in a way that DS cannot recognize? (2) How to prevent systems for breaching their promises? and (3) How to prevent the misuse of PHI after it has been released for secondary use?

Regulation and monitoring can give partial solution to first two challenges. Policy agents, self-destroying files, programmed death (apoptosis), destruction of cryptographic keys, and mutation engines have been proposed by researchers to give protection in the case of post-release [52,63]. The flexibility of developed architecture enables the DS to deploy any of these engines to control the secondary use of PHI.

In addition, there remain some more important challenges. The TC should understand both international and national regulations, and rules used by systems. Translation of narrative rules into machine-readable policies is an ongoing challenge [14]. The use of computer-understandable and context-aware polices requires either that all stakeholders accept a common policy language (such as Ponder, KAoS, Security Assertion Markup Language, eXtensible Access Control Markup Language, Rei, XPath-Based Preference Language, P3P, and APPEL) or that they use a method that enables semantically correct transformation between languages, based on ontologies [43,64,65]. Meta-policies such as P3P and Rei are candidates for the latter case [64,66,67]. In ubiquitous health, the use of a single policy language and a common ontology might be impossible. A possible solution is that the TC and the DS simply inform to systems about the ontology and policy language they use. If this is not possible, a service that maintains interoperability between policy languages and offers ontology reasoning should be developed [68]. In addition to policy, context and trust ontologies and other ontologies such as information and communication technology ontologies that describe systems’ architectural and organizational aspects and mechanisms are needed. Considering the future work, the authors will evaluate the architecture, and validate its feasibility and functionality in pilot setting. As a minimum, the proof of concept will be done. The authors will also demonstrate that the proposed solution is technically valid, safe to use, and efficient.

## Abbreviations

DS: data subject
PHI: personal health information
PII: personal identifiable information
TA: trust authority
TC: trust calculator
THEWS: Trusted eHealth and eWelfare Space

## References

[1] Ruotsalainen PS, Blobel BG, Seppälä AV, Sorvari HO, Nykänen PA (2012). A conceptual framework and principles for trusted pervasive health. J Med Internet Res.

[2] Bryce C, Dekker M, Etalle S, Le Metayer D, Minuer S (2007). Ubiquitous privacy protection. http://hal.inria.fr/inria-0039510.

[3] Westin AF (2003). Social and political dimensions of privacy. J Social Issues.

[4] Gambetta D (1988). Trust: Making and Breaking Cooperative Relations.

[5] Schoorman FD, Mayer RC, Davis JH (2007). An interactive model of organizational trust: past, present and future. Acad Manage Rev.

[6] Ruohomaa S, Kutvonen L, Herrmann P, Issarny V, Shiu S (2005). Trust management survey. Trust Management: Third International Conference, iTrust 2005, Paris, France, May 23-26, 2005, Proceedings.

[7] Abdul-Rahman A, Hailes S (2000). Supporting trust in virtual communities.

[8] Billhardt H, Hermoso R, Ossowski A, Conteno R (2007). Trust-based service provider selection in open environments.

[9] Bellotti V, Sellen A (1993). Design for privacy in ubiquitous computing environment. ECSCW '93: Proceedings of the Third European Conference on Computer-Supported Cooperative Work, 13-17 September 1993, Milano, Italy.

[10] Hong J, Ng DJ, Lederer S, Landay JA (2004). Privacy risk models for designing privacy sensitive ubiquitous computing systems. http://repository.cmu.edu/hcii/69.

[11] Berghe CV, Schunter M (2006). Privacy injector – automated privacy enforcement.

[12] Skinner G, Han S, Chang E, Hao Y, Jiming L, Wang Y, Cheung Y-m, Yin H, Jiao L, Ma J, Jiao Y-C (2005). A new conceptual framework within information privacy: meta privacy. Computational Intelligence and Security: International Conference, CIS 2005, Xi'an, China, December 15-19, 2005, Proceedings, Part II (Lecture Notes in ... Notes in Artificial Intelligence) (Pt. 2).

[13] Yan Z, Holtmanns S, Subramanian R (2007). Trust modeling and management: from social trust to digital trust. Computer Security, Privacy and Politics: Current Issues, Challenges and Solutions.

[14] Mont MC, Pearson S, Creese S, Goldsmith M, Papanikolaou N (2010). Towards a Conceptual Model for Privacy Policies - HPL-2010-82.

[15] Zwick D (1999). Models of Privacy in the Digital Age: Implications for Marketing and E-Commerce.

[16] Campbell R, Al-Muhtadi J, Naldurg P, Sampemane GM, Mickunas MD, Okada M, Pierce BC, Andre S, Hideyuki T, Yonezawa A (2002). Towards security and privacy for pervasive computing. Software Security - Theories and Systems : Mext-NSF-JSPS International Symposium, ISSS 2002, Tokyo, Japan, November 8-10, 2002.

[17] Abdul-Rahman A, Hailes S (1997). A distributed trust model.

[18] McKnight DH, Choudhury V, Kacmar C (2002). Developing and validating trust measures for e-commerce: an integrative typology. Inform Sys Res.

[19] Uddin GM, Zulkernine M, Ahmed SI (2008). Cat: a context-aware trust model for open and dynamic systems.

[20] Sabater J, Sierra C (2005). Review on computational trust and reputation models. Artif Intell Rev.

[21] Liu Z, Yau SS, Peng D, Yin Y (2008). A flexible trust model for distributed service infrastrucure.

[22] Ries S (2009). Trust in Ubiquitous Computing (PhD thesis).

[23] Becerra G, Heard J, Kremer R, Denzinger J (2007). Trust attributes, methods, and uses. http://citeseerx.ist.psu.edu/viewdoc/download?doi=10.1.1.100.6965&rep=rep1&type=pdf.

[24] Hussin Ab RC, Macaulay L, Keeling K (2007). The importance ranking of trust attributes in e-commerce Website. http://www.pacis-net.org/file/2007/1247.pdf.

[25] Lu Y, Weichao WW, Bhargava B, Xu D (2006). Trust-based privacy preservation for peer-to-peer data sharing. IEEE Trans Syst Man Cybern A.

[26] Almenarez F, Marin A, Campo C, Garcia C (2004). Managing ad-hOC trust relationships in pervasive computing environments. http://www.vs.inf.ethz.ch/events/sppc04/papers/sppc04_almenarez.pdf.

[27] Jameel H, Hung LX, Kalim U, Sajjad A, Lee S, Lee YK (2005). A trust model for ubiquitous systems based on vectors of trust values.

[28] Huang J, Nicol D (2009). A calculus of trust and its application to PKI and identity management.

[29] Krukow K, Nielsen M, Sassone V (2008). Trust models in ubiquitous computing. Philos Trans A Math Phys Eng Sci.

[30] Ray I, Ray I, Chakraborty S (2010). A context-aware model of trust facilitating secure ad hoc collaborations. Trust Modeling and Management in Digital Environments: From Social Concept to System Development.

[31] Mui L, Mohtashemi M, Halberstadt A (2002). A computational model of trust and reputation for e-business.

[32] Bertino E, Ferrari E, Squicciarini A (2004). Trust-X: a peer-to-peer framework for trust establishment. IEEE Trans Knowl Data Eng.

[33] Chakraborty S, Ray I (2007). p-Trust: a new model of trust to allow finer control over privacy in peer-to-peer framework. J Comput.

[34] Lederer S, Mankoff J, Dey AK, Beckmann CP (2003). Report No UCB/CSD-3-1257.

[35] Friedewald M, Vildjiounaite E, Punie Y, Wright D (2007). Privacy, identity and security in ambient intelligence: a scenario analysis. Telematics Informatics.

[36] Adams A, Sasse MA (2001). Privacy in multimedia communications: protecting users, not just data. http://www.eis.mdx.ac.uk/ridl/aadams/hci01.pdf.

[37] Jiang X, Landay AJ (2002). Modeling privacy control in context-aware systems. IEEE Pervasive Comput.

[38] Kapadia A, Henderson T, Fielding J, Kotz D, LaMarca A, Langheinrich M, Truong KN (2007). Virtual walls: protecting digital privacy in pervasive environments. Pervasive Computing: 5th International Conference, PERVASIVE 2007, Toronto, Canada, May 13-16, 2007, Proceedings (Lecture Notes in Computer Science / Information ... Applications, incl. Internet/Web, and HCI).

[39] Diaz C, Seys S, Claessens J, Preneel B (2003). Towards measuring anonymity. Privacy Enhancing Technologies: Second International Workshop, PET 2002, San Francisco, CA, USA, April 14-15, 2002: revised papers.

[40] Lederer S, Mankoff J, Dey AK (2002). Report No, UCB/CSD-2-1288.

[41] Dritsas S, Gritzalis D, Lambrinoudakis C (2006). Protecting privacy and anonymity in pervasive computing: trends and perspectives. Telematics Informatics.

[42] Gray E, O’Connell P, Jensen C, Weber S, Seigneus JM, Yong C (2002). Technical Report 66.

[43] Kagal L, Berners-Lee T, Connolly D, Weitzner D (2008). Promoting Interoperability Between Heterogeneous Policy Domains.

[44] Skopik F (2010). Dynamic Trust in Mixed Service-oriented Applications (dissertation).

[45] Joshi A, Finin T, Kagal L, Parker J, Patwardhan A (2008). Security policies and trust in ubiquitous computing. Philos Trans A Math Phys Eng Sci.

[46] Khiabani H, Sidek ZM, Manan JL (2010). Towards a unified trust model in pervasive systems.

[47] Salah H, Eltoweissy M, Abel-Hamid A (2008). Computational Trust for Peer-to-Peer Web Services.

[48] Pearson S, Mont MC (2011). Sticky policies: an approach for managing privacy across multiple parties. Computer.

[49] Wang Y, Kobsa A, Grupta M, Sharman R (2008). Privacy-enhancing technologies. Handbook of Research on Social and Organisational Liabilities in Information Security.

[50] CEN (2005). Analysis of Privacy Protection Technologies, Privacy-Enhancing Technologies (PET), Privacy Management Systems (PMS) and Identity Management Systems (IMS), the Divers Thereof and the Need for Standardization.

[51] Lilien L, Bhargava B (2006). A scheme for privacy-preserving data dissemination. IEEE Trans Syst Man Cybern A.

[52] Tschudin C, Vitek J, Jensen C (1999). Apoptosis - the programmed death of distributed services. Secure Internet Programming: Security Issues for Mobile and Distributed Objects, LNCS.

[53] Pallapa G, Kumar M, Das SK (2007). Privacy infusion in ubiquitous computing.

[54] Kahl C, Böttcher K, Tschersich M, Heim S, Rannenberg K, Rannenberg K, Varadharajan V, Weber C (2010). How to enchance privacy and identity management for mobile communities: approach and user drive concepts of the PICOS project. Security and Privacy - Silver Linings in the Cloud: 25th IFIP TC 11 International Information Security Conference, SEC 2010, Held as Part of WCC 2010, Brisbane, Australia, September 2010 Proceedings.

[55] HL7 International, Inc (2010). HL7 Privacy, Access and Security Services (PASS) Specification.

[56] Hoaglund JA (2000). Specifying and Implementing Security Policies Using LaSCO, the Language for Security Constraints on Objects (PhD dissertation).

[57] Price BA, Adam K, Nuseibeh B (2005). Keeping ubiquitous computing to yourself: a practical model for user control of privacy. Int J Hum-Comput St.

[58] Patwardhan A, Korolev V, Kagal L, Joshi A (2004). Enforcing policies in pervasive environments.

[59] Grimm S, Lamparter S, Abecker A, Agarwal S, Eberhart A (2004). INFORMATIK 2004 – Informatik verbindet, Band 2, Proceedings of Semantic Web Services and Dynamic Networks.

[60] Cote PP (2004). PC Expressions.

[61] Gao F, He J, Ma S (2012). Trust based privacy protection method in pervasive computing. J Netw.

[62] Louviere JJ, Hensher DA, Swait JD (2000). Stated Choice Methods: Analysis and Applications.

[63] Zuo Y, O'Keefe T (2007). Post-release information privacy protection: a framework and next-generation privacy-enhanced operating system. Inf Syst Front.

[64] Kumaraguru P, Cranor LF, Lobo J, Calo SB (2007). A survey of privacy policy languages. Workshop on Usable IT Security Management (USM 07).

[65] Damianou N, Bandara A, Sloman M, Lupu EC (2002). A Survey of Policy Specification Approaches, Technical Report.

[66] Karjot G, Schunter M, Waidner M (2003). Platform for enterprise privacy practices: privacy-enabled management of customer data.

[67] Kagal L, Finin T, Joshi A (2003). A policy language for a pervasive computing environment.

[68] Blobel B (2011). Ontology driven health information systems architectures enable pHealth for empowered patients. Int J Med Inform.

